# Supplementary material for: Patterns of Intron Gain and Loss in Fungi
Source: PLoS Biol. 2004 Nov 30;2(12):e422. doi: 10.1371/journal.pbio.0020422 (PMC532390; doi:10.1371/journal.pbio.0020422)
Supplement: Table S1 — Also available at http://genes.mit.edu/NielsenEtAl/. (4.3 MB ZIP). [file pbio.0020422.st001.zip › NielsenEtAl/html/63.html]

AN3666.1.NCU02631.1.MG04434.1.FG01007.1


```
 CLUSTAL W (1.82) Multiple Sequence Alignments - Introns Inserted


Sequence 1: MG04434.1	215 aa
Sequence 2: FG01007.1	212 aa
Sequence 3: NCU02631.1	216 aa
Sequence 4: AN3666.1	223 aa
Alignment Length: 225 aa
Number Identitical Residues: 135 aa
Alignment Score (without introns) 6171


MG04434.1 	--MYGDVGNKL0VQHAKRTQNLTHLPPYQTELVRAATREVRDLNRDVTSLLEPFEGAFDP
NCU02631.1	MPMYGDLGNKL0VQHAKRTQNLSHLPPYQTELVRAVAREIRDLDKDVASLLEPFQGSFDP
FG01007.1 	--MYGDLGNKL0VQHAKRTQNLAHLPPYQTEIVRAVTREVRDLDKDVGELLEPFQGSFDP
AN3666.1  	--MYGELGNKL0VQHAKRTQSLAHLPPYQTELVRTVAREVRDLDRDLAHLLAPFEGSFNP
          	  ***::**** ********.*:********:**:.:**:***::*:  ** **:*:*:*

MG04434.1 	NEDKATACALLVNHLSMRRNKRCLLAYHRTRTDKLEELVWSGADVMELSAQ------GQQ
NCU02631.1	SAEQATACTLLVNHLSMRRNKRCLLAYHRIRTDKLEELVWNGADILDLA--------GQT
FG01007.1 	SADQAVSCTLLVNHLSMRRNKRCLLAYHRTRTDKLEEQVWNGSDVVDLS--------GQQ
AN3666.1  	SQEPAVACALLVDHLCIRRNKRCLLAYHRVRTEKLEELCWKGIDVLEHQQATAAGDDGGQ
          	. : *.:*:***:**.:************ **:****  *.* *:::    :::...*  

MG04434.1 	LRDGSGGGPGP--ADGPTGGLSPQEEDYVRQYSDLLAAYKGQWTDVDLTGSLEPPRDLFI
NCU02631.1	TGGPKGVTEGNE-GGGTTSSLSPQEEDYFRQFGDLLALYKGQWTDIDLTGSLEPPRDLFI
FG01007.1 	VRDTSNSGTG---SSGATSSLSPQEEEYVRQYSDLLAAYKGQWTDIDLTGSLEPPRDLFI
AN3666.1  	MGGQNSESTGAQGTSGNESSLSPEEEEYFREYSDMLATYKGQWTDIDLTGSLEPPKDLFI
          	  . ..   * .. .*  ..***:**:*.*::.*:** *******:*********:****

MG04434.1 	DVRVLKDAGEIQTEYG2AITLSKNSQFYVRQGDVERLIAQGYLQKLS
NCU02631.1	DVRVLKDAGEIQTEYG2AINLTKNSQFYVRQGDVERLIVQGYLQKLG
FG01007.1 	DVRVIKDAGEIQTEYG2AITLTKNSQFYVRQGDVERLITQGYLHKLG
AN3666.1  	DVRVLKDAGEIQTEYG2VINLTKNSQLYVRHGDVERLIAQGFLEQLS
          	****:*********** .*.*:****:***:*******.**:*.:*.
```
